# Frameless linac-based stereotactic radiosurgery (SRS) for brain metastases: analysis of patient repositioning using a mask fixation system and clinical outcomes

**DOI:** 10.1186/1748-717X-6-158

**Published:** 2011-11-16

**Authors:** Giuseppe Minniti, Claudia Scaringi, Enrico Clarke, Maurizio Valeriani, Mattia Osti, Riccardo Maurizi Enrici

**Affiliations:** 1 Department of Radiation Oncology, Sant' Andrea Hospital, University Sapienza, Rome, Italy; 2 Department of Neuroscience, Neuromed Institute, Pozzilli (IS), Italy

**Keywords:** stereotactic radiosurgery, positioning reproducibility, isocenter verification, brain metastases

## Abstract

**Purpose:**

To assess the accuracy of patient repositioning and clinical outcomes of frameless stereotactic radiosurgery (SRS) for brain metastases using a stereotactic mask fixation system.

**Patients and Methods:**

One hundred two patients treated consecutively with frameless SRS as primary treatment at University of Rome Sapienza Sant'Andrea Hospital between October 2008 and April 2010 and followed prospectively were involved in the study. A commercial stereotactic mask fixation system (BrainLab) was used for patient immobilization. A computerized tomography (CT) scan obtained immediately before SRS was used to evaluate the accuracy of patient repositioning in the mask by comparing the isocenter position to the isocenter position established in the planning CT. Deviations of isocenter coordinates in each direction and 3D displacement were calculated. Overall survival, brain control, and local control were estimated using the Kaplan-Meier method calculated from the time of SRS.

**Results:**

The mean measured isocenter displacements were 0.12 mm (SD 0.35 mm) in the lateral direction, 0.2 mm (SD 0.4 mm) in the anteroposterior, and 0.4 mm (SD 0.6 mm) in craniocaudal direction. The maximum displacement of 2.1 mm was seen in craniocaudal direction. The mean 3D displacement was 0.5 mm (SD 0.7 mm), being maximum 2.9 mm. The median survival was 15.5 months, and 1-year and 2-year survival rates were 58% and 24%, respectively. Nine patients recurred locally after SRS, with 1-year and 2-year local control rates of 91% and 82%, respectively. Stable extracranial disease (P = 0.001) and KPS > 70 (P = 0.01) were independent predictors of survival.

**Conclusions:**

Frameless SRS is an effective treatment in the management of patients with brain metastases. The presented non-invasive mask-based fixation stereotactic system is associated with a high degree of patient repositioning accuracy; however, a careful evaluation is essential since occasional errors up to 3 mm may occur.

## Introduction

Stereotactic radiosurgery (SRS) has become increasingly used for treatment of patients with brain metastases. Its efficacy when used alone or in combination with whole brain radiation-therapy (WBRT) has been demonstrated in several randomized trials and multi-institutional studies [1-5].

SRS has traditionally been performed using an invasive fixed head ring that establishes the stereotactic coordinates of the target and allows for an accuracy of immobilization and positioning less than 1 mm during image acquisition and treatment. More recently, as an alternative to the invasive patient fixation technique, different frameless stereotactic systems have been implemented. A variable positioning accuracy of 1-4 mm has been reported for frameless stereotactic systems [6-14], reflecting, at least in part, different methods in patient fixation, positioning, and assessment of accuracy. The use of small margin of safety that must be added to the target volume for errors in localization and set-up is essential in order to minimize the potential treatment-related complications of SRS. Volumes of normal brain receiving high dose of radiation are in fact predictive of the development of brain radionecrosis, which is reported in up to 47% of treated lesions for brain volumes larger than 10 cc receiving a dose of 12 Gy [15].

Only limited data on tumor control and target localization have been provided specifically using linac-based frameless devices. In this study, we report our clinical experience in patients with brain metastases with the use of a commercially available frameless SRS system. In addition accuracy of target positioning was evaluated using repeat computed tomography (CT) images.

## Patients and Methods

One hundred two patients treated consecutively with frameless SRS as primary treatment at University of Rome Sapienza Sant'Andrea Hospital between October 2008 and April 2010 and followed prospectively were involved in the study. Patient characteristics are listed in Table 1. Sixty-four patients were treated for 1 metastasis, 24 patients for 2 metastases, and 14 patients for 3 metastases. The median age at the time of SRS was 64 years (range 26-81). The most common histologies were lung, breast, and melanomas. The most common location was parietal lobe followed by frontal and temporal lobe. According to RTOG recursive partitioning analysis (RPA) classes for brain metastases, 32 patients (31.5%) were in RPA Class I, 58 patients (57%) in RPA Class II, and 12 (11.5%) patients in RPA Class III. Patients were examined clinically one month after SRS and then every 2 months. MRI was made every 2 months in the first year after the treatment, and then every 3 months or as appropriate according to the neurological conditions. The size of treated lesions was measured in three dimensions. Complete and partial response were defined as total radiographic disappearance of lesion or decrease in tumor volume > 50%. Local progression was defined as radiographic increase in the size of metastatic lesion.

**Table 1:** Summary of tumor charaterictiscs and treatment parameters of patients treated with radiosurgery

	No (%)
***Number of patients***	102
***Median age***	64
***Sex (F/M)***	52/50
***No of lesions per patient***	
1 lesion	64 (63%)
2 lesions	24 (23%)
3 lesions	14 (14%)
***Histology***	
lung	54 (53.5%)
breast	17 (16.5%)
melanoma	14 (13.5%)
others	17(16.5%)
***Tumor location***	
frontal	31 (20%)
parietal	37 (24%)
temporal	30 (19%)
cerebellar	23 (15%)
occipital	26 (17%)
brainstem	7 (5%)
***Radiosurgical dose***	
20 Gy	86 (56%)
18 Gy	44 (28%)
15-16	24 (16%)
***Tumor volume (cm^3^)***	
median	1.6
range	0.03-16.3
***Treated volume (cm^3^)***	
median	2.2
range	0.2-18.8

### SRS procedure

After obtained informed consent, patients underwent contrast-enhanced T1-weighted magnetic resonance imaging (MRI) (26 cm FOV, 512 × 512 pixel size, 1 mm slice interval) using a 1.5 Tesla MRI (Siemens Sonata, Siemens Medical Systems, Erlangen, Germany). Patient immobilization was achieved by using the commercially available BrainLab head mask fixation system. In addition, a mouth bite positioned against the upper dentition attached to the stereotactic frame was applied to prevent any head tilt movement. The characteristics of the system have been previously described [16]. Before the CT localization a localizer box was mounted to the BrainLAB mask system in order to provide a three-dimensional (3D) stereotactic coordinate array for target localization. During the procedure the patient was laid on the CT couch with the system secured onto a custom-made platform. CT imaging was performed using the GE 16-slice scanner. CT (General Electric Medical System) scanning was done in spiral mode using a pitch of 0.75, 512 × 512 pixel size, and slices in thickness and spacing of 1.2 mm acquired throughout the entire cranium. Tube voltage and tube potential were set at 130 kV and 300 mA to obtain high quality reconstructed slices.

The MRI and planning CT datasets were imported into the BrainLab planning system and stereotactic coordinates localization were performed by the software by identifying the location of six localizer rods on the outside surfaces of the right, left, and anterior walls of the localizer box. Localization establishes the 3D stereotactic coordinate system for treatment planning and delivery. The target volume was identified on the basis of the fused CT and magnetic resonance (MR) images. The gross tumor volume (GTV) was delineated as a contrast-enhancing tumor demonstrated on MRI scans. CTV was considered the same as GTV. The planning target volume (PTV) was generated by the geometric expansion of GTV plus 1.0 mm. Radiosurgical dose was 20 Gy for metastases with a volume ≤ 4.3 cm^3 ^(corresponding to a sphere of 2 cm in diameter), 18 Gy for metastases with a volume of 4.3-14.1 cm^3^, and 16 Gy for metastases with a volume > 14.1 cm^3^. Doses were prescribed to the 80-90% isodose line normalized to the maximum dose. All radiation doses were delivered in a single fraction with 6-10 noncoplanar dynamic arcs by using a 6-MV LINAC. Patients with multiple lesions often underwent treatment in several sessions over several days.

Immediately before treatment, all patients underwent CT verification to check the accuracy of isocenter position [16]. Firstly, the CT verification set was imported in the planning system and localized automatically by the planning software through identification of the stereotactic fiducials in the same way as for planning CT. Since this step spatially co-registers the stereotactic coordinate systems of planning CT and verification CT with the respect to the localizer box, errors in patient repositioning result in a shift of anatomical isocenter position. In the second step the planning CT and the CT verification were fused. Following fusion, anatomy was co-registered. Since all brain structures were spatially matched, any translation of isocenter position due to patient repositioning error resulted in a mismatch of the localizer rods of the localizer box. As consequence, the 3D stereotactic coordinates of isocenter in the verification CT changed accordingly. Finally, the new isocenter coordinates were recorded, and the isocenter shift between verification and planning CT calculated.

For deviations more than 1 mm the treatment was replanned on the basis of the new isocenter coordinates. The whole procedure including verification of isocenter and replanning lasted less than 8 minutes. During this time patients fitted with the mask were gently and slowly moved to a wheelchair and transported from CT simulation room to the treatment room, and positioned on the LINAC treatment couch. A post-treatment CT was performed in 60 patients. Differences in isocenter position calculated by planning CT and post-treatment CT fusion were assumed to serve as an indication of the stability on the patient's head within the mask during treatment (intra-fraction motion) and transportation.

Local control and survival from the date of SRS were calculated using the Kaplan-Meier. Deviations of isocenter coordinates in each direction were measured as mean ± standard deviation (SD) for all patients. The 3D displacement determined by the square root of the sum of squares of the displacements seen in the 3 directions was calculated. Analysis of subgroups was performed using the log-rank test, and p = 0.05 was the criterion for statistical significance.

## Results

### Accuracy of positioning

The relocation accuracy of the isocenter determined from CT verification before the treatment is shown in Table 2. The mean measured isocenter displacements were 0.12 mm (SD 0.35 mm) in the lateral direction, 0.2 mm (SD 0.4 mm) in the anteroposterior, and 0.4 mm (SD 0.6 mm) in craniocaudal direction. The maximum displacement of 2.1 mm was seen in craniocaudal direction. The mean 3D displacement was 0.5 mm (SD 0.7 mm), being maximum 2.9 mm. A 3D displacement more than 1 mm was seen in 31 metastases (20%), being more than 2 mm in 7 metastases (4.5%), and requiring treatment replanning. There was a correlation between deviation of isocenters and the position of metastases in the brain, with the maximum 3D displacement observed for metastases located in the cerebellar and frontal lobes (cerebellar/frontal lobes versus other lobes, p = 0.02).

**Table 2:** Accuracy of isocenter relocation at CT verification

Direction (mm)	Mean	SD	Range
Cranio-caudal	0.4	0.6	-1,2 - 2.1
Medio-lateral	0.12	0.35	-0.9 - 1.4
Anterior-posterior	0.2	0.4	-1,6 - 1.3
3D-displacement	0.5	0.7	0.1-2.9

SD, standard deviation

A post-treatment CT was made in 60 patients (Table 3). Patients fitted with the mask were transported in a wheelchair from the treatment room to the CT room. The mean measured isocenter displacements were 0.04 mm (SD 0.14 mm) in the lateral direction, 0.06 mm (SD 0.15 mm) in the anteroposterior direction, and 0.08 mm (SD 0.2 mm) in craniocaudal direction. The mean 3D displacement was 0.09 mm (SD 0.28 mm), with the maximum shift of 0.6 mm.

**Table 3:** Mean and standard deviation of isocenter displacement between CT verification and post-treatment CT

Direction (mm)	Mean	SD	Range
Cranio-caudal	0.08	0.2	-0.3 - 0.2
Medio-lateral	0.04	0.14	-0.3 - 0.2
Anterior-posterior	0.06	0.15	-0.5 - 0.4
3D-displacement	0.09	0.28	0-0.6

SD, standard deviation

Quality control procedures at the CT scanner, simulation room and linear accelerator were routinely performed. The accuracy of coincidence of the radiation isocenter of the treatment unit and the laser-defined room coordinate system for patient alignment (TC scanner, simulator and treatment rooms) resulted within 0.8 mm.

### Outcome

**Figure 1:**
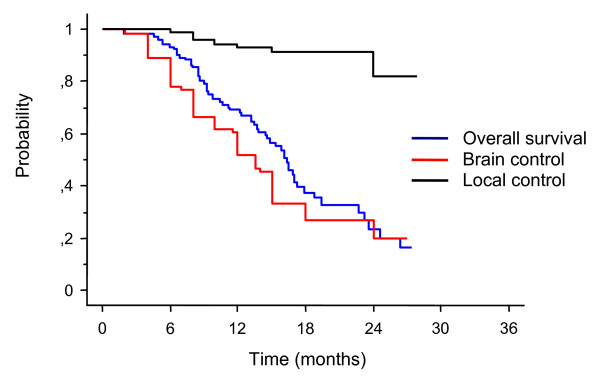
**Kaplan-Meier analysis of overall survival, brain control, and local control**.

At a median clinical follow-up of 13.5 months (range 2-32 months) median survival and brain control were 15.5 months and 12 months, respectively (Figure 1). The 1-year and 2-year survival rates were 67% and 20%, and respective brain control rates were 50% and 21%. Forty-three percent of patients succumbed to their extracranial disease and 19% died of progressive intracranial disease. Data were reported to July 2011. At this time 38% of patients were alive. Intracranial tumor progression was observed in 60 patients. The 12-month and 24-month actuarial rates of developing new brain metastases were 43% and 74%, respectively. Nine patients recurred locally after SRS. The 1-year and 2-year local control rates were 91% and 82%, respectively. Salvage WBRT was applied in 29 patients and further SRS in 30 patients. Thirty-seven metastases (24%) had a complete response, 59 (38%) had a partial response, and 49 (32%) remained stable. A clinical neurological improvement of pre-SRS existing symptoms was recorded in 18 out of 57 patients (31%) following SRS.

Extracranial disease (P = 0.0001), KPS (P = 0.001), number of metastases (P = 0.01), and RPA class (P = 0.0001) were predictive factors for survival. On multivariate analysis stable extracranial disease (P = 0.001) and KPS > 70 (P = 0.01) were associated with the most significant survival benefit. Stable extracranial disease (P = 0.001), KPS > 70 (P = 0.01), and number of metastases (1 vs > 1, P = 0.001) were significant predictive factors for brain control; however, only extracranial disease (P = 0.006) and number of metastases (P = 0.005) were independent predictors on multivariate analysis. No significant prognostic factors were associated with local control, however there was a trend toward worse control for melanoma histology (p = 0.15).

Brain radionecrosis, as suggested by MR imaging or confirmed by histology (n = 9) occurred in 39 (25%) out of 154 treated lesions. Radionecrosis was symptomatic in 15 patients, being associated with severe neurological complications (RTOG Grade 3 and 4) in 7 patients.

## Discussion

An essential prerequisite of a frameless stereotactic system is that patient immobilization and positioning are performed with a high degree of accuracy in order to deliver a safe therapeutic radiation dose as for invasive frame-based SRS. Different frameless stereotactic systems, including infrared camera guidance [17], dental [18-20], implanted fiducial markers [21,22], and mask fixation system [6-12] have been developed in the last two decades. In our study using a mask-based stereotactic system we have evaluated the accuracy of isocenter relocation by repeat CT scans. Mean and SD of displacements for each direction were 0.1 mm (SD 0.35 mm) in the mediolateral direction, 0.2 mm (SD 0.4 mm) in the anteroposterior direction, and 0.4 mm (SD 0.6 mm) in the craniocaudal direction. The mean 3D displacement was 0.5 mm (SD 0.7 mm), being maximum 2.9 mm. Using a similar stereotactic mask fixation system Wong et al. [11] reported a mean and maximum 3D displacements at the isocenter evaluated by CT verification of 0.7 and 2.5 mm, respectively. Fuss et al [13] in a series of 22 patients with 43 cranial lesions have reported a mean 3D target isocenter translation of 1.64 ± 0.84 mm, and a maximum dislocation of 3.39 mm, and similar results have been shown by others [7-10].

In our study repeat CT scan with a thickness of 1.2 mm and standard high-resolution imaging as the matrix for data acquisition was used to evaluate the accuracy of isocenter relocation. Analysis of repeated CT datasets has the advantage of high resolution imaging as compared with portal films [13], although a clear limit of our procedure is that it can not offer data on real repositioning accuracy on the treatment table. Recent development of image-guided frameless radiosurgery systems include the use of optical image guidance and X-ray to evaluate patient repositioning with an accuracy of the system similar to that reported for invasive frames [23-28]

An isocenter displacement > 1 mm was found in approximately 20% of treated lesions, being more than 2 mm in 4.5% of lesions. Although a margin from GTV to PTV expansion of 3 mm could compensate the inaccuracy of positioning reproducibility reported in our series, this will increase the volume of normal brain treated at high doses (up to 3 times for a lesion of 1.6 cm^3 ^corresponding to our median tumor volume) and would likely be unacceptable to avoid serious treatment-related complications. Thus, in such patients the treatment was replanned according to new isocenter coordinates as calculated on the basis of CT verification. The time required for the CT verification was approximately 5 minutes. Another 7 minutes were required for image transfer, identification of the rods, fusion, recalculation of isocenter, and replanning. Our verification method allows us to use an expansion from GTV to PTV of 1 mm during the planning, and this may have important clinical implications. Several studies have in fact shown a significant correlation between normal brain volume receiving a dose of 12 Gy and the development of radionecrosis in patients treated with SRS for brain metastases [15,29]. In a series of 310 brain metastases treated with SRS at our institution the actuarial risk of brain radionecrosis at 1 year was up to 47% for volumes of brain larger than 10.9 cm^3 ^treated at a dose of 12 Gy, and similar results have been reported by others [29]. In our current clinical practice the reported procedure permits the use of strict margins for SRS while maintaining an appropriate coverage of the target, and possibly avoiding serious treatment-related complications.

The intra-fraction motion is of concern during frameless SRS. In order to evaluate the motion of the patient's head during the radiosurgical procedure, a post-treatment CT was performed in 60 patients. The differences in isocenter shift calculated by fusing the verification CT and post-treatment CT represent an indication of the accuracy of patient's head immobilization during either treatment or transportation from CT couch to the treatment room. The absence of significant movements during the different steps of the whole procedure confirms the excellent stability of our mask-based frameless systems and justifies its use for SRS.

Because the ultimate validity of a procedure is measured in terms of clinical results, we have examined the local control as the most sensitive clinical outcome for assessing target accuracy for brain metastases treated with frameless SRS. The tumor control of 91% at 12 months and 82% at 24 months is in the best range reported using other frameless stereotactic systems [30-32], and confirms that frameless SRS is a viable option for patients with brain metastases with an outcome similar to that observed following frame-based SRS [33-37]. Certainly, frameless SRS has several advantages compared with traditional frame-based techniques including patient comfort, greater flexibility in scheduling treatment planning and treatment procedure, possibility to treat multiple lesions in different days without the need to reapply a head frame, and the ability to use "multisession radiosurgery" to treat large lesions.

Our study has some limitations. Patient relocation evaluated by comparison of localization and verification CT scans does not include errors which are related to the treatment unit as laser alignment, machine and couch accuracy. Thus, although a margin of 1 mm was associated with an excellent local control and accepatable toxicity, large series and longer follow-up need to confirm the results reported in our series.

In conclusion, the results presented in this study confirm the high accuracy of patient repositioning with the use of our non-invasive mask-based fixation stereotactic system. However, a careful evaluation of the reproducibility of patient head position in the mask is essential since occasional setup errors up to 3 mm may occur. The promising results in terms of local control and survival support the use of linac-based frameless SRS as a common technique in the management of patients with brain metastases.

## Conflict of interests

The authors declare that they have no competing interests.

## Authors' contributions

GM conceived of the study, participated in its design and coordination, and drafted the manuscript. CS and EC carried out the radiosurgical procedures, and participated in analysis and interpretation of data. MV and MFO participated in analysis of data and helped to draft the manuscript. RME critically reviewed/revised the article. All authors read and approved the final manuscript.
